# Feasibility analysis and study of an intrahepatic portal vein infection hepatic alveolar echinococcosis C57 mouse model

**DOI:** 10.3389/fvets.2022.994652

**Published:** 2022-12-15

**Authors:** Weili Tian, Wenchao Ji, Jun Li, Wenya Liu, Zhi Wen, Juan Wu

**Affiliations:** ^1^Imaging Center, The First Affiliated Hospital of Xinjiang Medical University, Ürümqi, China; ^2^The Affiliated Tumour Hospital of Xinjiang Medical University, Ürümqi, China

**Keywords:** liver, alveolar echinococcosis model, comparison of tissue appearance, histopathology, hepatic alveolar spines ball larva disease

## Abstract

**Objective:**

The aim of the study was to establish and study an intrahepatic portal vein infection hepatic alveolar echinococcosis (HAE) C57 mouse model and provide a theoretical basis for clinical research on HAE.

**Methods:**

C57 mice were used to establish the HAE mouse model. The location, size, morphology, appearance, and pathological changes in liver lesions in different groups of mice were characterized using ultrasound, magnetic resonance imaging (MRI), and haematoxylin and eosin staining.

**Results:**

The mortality rate of the C57 mice was 20%, and the success rate of infection was 75%. The abdominal ultrasound images and MRIs clearly indicated the location, size, shape, and appearance of the liver lesions and the relationship between the lesions and the adjacent organs. The size, morphology, and signal of the livers in the control group were normal. The pathological results of the experimental group indicated a hepatic vesicular acinar cyst, while those of the control group exhibited normal livers.

**Conclusion:**

The intrahepatic portal vein infection HAE mouse model was successfully established.

## Introduction

The genus *Echinococcus*, commonly known as tapeworm, includes the following three species: *Echinococcus multilocularis, Echinococcus vogeli*, and *Echinococcus granulosus* (1). Hydatid disease is an uncommon parasitic infection, caused mainly by the larval form of *Echinococcus granulosus*, and less frequently by multiatrial parasitic infection, which mainly causes hepatic vesicular echinococcosis (2). Hepatic alveolar echinococcosis (HAE) is a relatively rare but serious parasitic infectious disease that can be life-threatening (3). In this study, the lesion grows slowly, which is similar to some liver tumors. The disease can be found in both men and women in the northern hemisphere, with no significant difference in the infection rates between the sexes. According to the existing literature, the peak age is between 50 and 70 years (4, 5). The natural course of the disease begins with an asymptomatic incubation period of approximately 5–15 years, followed by a chronic process. Lesions are inadvertently discovered in more than one-third of patients during a physical examination (6).

The growth and development of hydatid cyst tissue *in vivo* can eliminate the interaction between the host and the parasite and other factors, which is an ideal method of preserving a hydatid cyst. Relevant studies demonstrated that it is feasible to establish *in vitro* models of *Echinococcus multilocularis* using the supernatant of liver cancer, colorectal cancer, and cervical cells (7). Main clinical diagnostic methods for hepatic alveolar hydatid cysts include ultrasound, computerized tomography (CT), magnetic resonance imaging (MRI), and positron emission tomography/CT scanning. Main treatment methods for hepatic alveolar hydatid cysts include benzene and imidazole derivatives, percutaneous drainage, and surgical resection. Liver transplantation should be considered only as a last resort. If left untreated, the disease could lead to liver failure and even death (8). Therefore, early detection and treatment are crucial.

One study reported that C57 mice are the most suitable model for establishing HAE (8). Thus, to improve the overall understanding of HAE, 20 C57 mice were selected to establish an intrahepatic portal vein infection model, and ultrasound and MRI manifestations and pathological HAE results were compared between the experimental and control mice. The model is simple and easy to use, provides a basis for clinical experimentation, and has a high success rate.

## Materials and methods

### Main instruments and reagents

The main instruments for detection were an ultrasonic system (Mindray, China, KM431), a small animal nuclear magnetic resonance (NMR) imager (BS-70), and a magnetic resonance spectrometer superconducting magnet system (Biospec 70/20 USR, Brock, Germany). The reagents used were normal saline (MDL: MFCD00003477, Sigma), ampicillin (S414801, Selleck), isoflurane (YZ-1349014, Solarbio), and iodophor (210707162457, Klamar).

### C57 mice

In this study, 40 C57 mice (clean grade, weight 17–22 g, 9–10 weeks old) were selected. A total of 20 C57 mice were selected as the experimental group, and 20 mice were allocated to the control group. The animals were purchased from the Animal Experiment Center, First Affiliated Hospital of Xinjiang Medical University and were cage-fed. The laboratory is accredited by the International Association for Evaluation and Accreditation of Laboratory Animal Care. The animal experiments were approved by the Laboratory Animal Management Committee of First Affiliated Hospital of Xinjiang Medical University (approval no. 20190225-108).

The laboratory mice were brought into the laboratory and kept for a week to acclimatize. The laboratory temperature was 20 ± 5°C, and the relative humidity was 50 ± 3%, varying 6–8 times per hour for 12 h. The 40 C57 mice were raised in cages with labeled cage cards and were regularly given food and water but were free to eat and drink when they liked. The rats' general conditions, including mental state, diet, defecation, and body movement, were observed daily.

### Preparation of the *Echinococcus* suspension

*Echinococcus multilocularis* protoscoleces were obtained from the intraperitoneal lesions of BALB/c mice under aseptic conditions (9). Following anesthesia, the breeder mice were killed, and the intraperitoneal *Echinococcus multilocularis* tissue was removed under sterile conditions and rinsed three times with an aseptic phosphate-buffered saline (PBS) solution. The tissue was cut, ground, and sieved, and the precipitate was then rinsed three times with a sterile PBS solution until the suspension was milky white. The suspension was prepared using aseptic PBS at a concentration of 20%. Finally, 0.1 g of ampicillin was added to the suspension to remove any bacteria and prepare for inoculation. The suspensions were prepared with sterile PBS at 20% concentration, and 0.1 g of ampicillin was again added to the suspension to remove bacteria.

### Mouse model of *Echinococcus multilocularis* infection

The mice were fasted for 12 h prior to surgery and water-restricted for 1 h prior to anesthesia. The mice were anesthetized using a specialized anesthesia machine and isoflurane. Following successful anesthesia, the mice were immobilized on a surgical board. Their abdomens were sterilized with medical iodine and were then incised along the midline to expose the intrahepatic portal vein. In the experimental group, the prepared *Echinococcus* suspension (2,000 pieces/piece) was injected into the intrahepatic portal vein *via* a syringe. We avoided injecting a large amount of fluid into the mouse circulatory system in a short period of time to prevent acute heart failure. Finally, the abdomen was closed following compression and haemostasis, and the mice were placed back in their cages for rearing after recovery on the heating table. The mice in the control group were injected with an equal volume of normal saline following the surgery.

### Verification

Mindray equipment (China, 12345) was used for ultrasound detection. The abdomen was selected as the scanning location, and the indicators of the instrument were adjusted to achieve an optimum state. The mice were anesthetized prior to the scan and immobilized in a supine position following successful anesthesia, and the abdominal ultrasound scan was then performed. A 2-cm-diameter probe was used, operating at a frequency of 1–5 MHz with a swept gain rate of 1–8 dB/cm. The scans were usually performed during respiratory inspiration, with the scanning time of approximately 10 s and a pulse repetition frequency of 150 Hz, with the two-dimensional P-scans containing ~1,500 lines each.

For MRI scanning, a small animal NMR (BS-70) magnetic resonance spectrometer superconducting magnet system (Biospec 70/20 USR, Bruck, Germany) was used, with a magnetic field intensity of 7 T, a transparent aperture of 200 mm, a stray field (5 Gauss) of +/ – 1.5 m axial and +/ – 1.5 m radial, a length of 1.312 m, and a diameter of 1.12 m. The mice were anesthetized prior to the examination, and were fixed in a supine position for the MRI scanning.

Liver pathology was determined using haematoxylin and eosin (H&E) staining, for which the C57 mice infected with vesicular echinococcosis were killed. The liver tissues of the mice were fixed in a 10% neutral phosphate-buffered formalin solution before being dehydrated and embedded in paraffin to produce conventional paraffin sections. The sections were cut into 4-μm-thick pieces and stained with H&E. The histopathological changes in the liver tissues were observed under an optical microscope. The pathological results of the two groups were then compared.

### Statistical analysis

The enumeration data were analyzed using SPSS13.0 software. In this study, the count data were expressed in terms of number (%). The characteristics and homogeneity of the mice within the groups were examined using the analysis of variance method. A *P*-value of >0.05 indicated that the difference in the mouse characteristics was not significant.

## Results

### Statistics on the infection rate of the mice

In this study, 20 C57 mice were allocated to the control group, and 20 were assigned to the experimental group. No significant difference was observed in the characteristics of the mice in the experimental group (Table 1). Also, in the experimental group, four mice died during anesthesia and operation, with a mortality rate of 20%. Of the remaining 16 mice, 12 were successfully infected, with a success rate of 75%.

**Table 1:** Variance analysis of characteristics of mice.

**Number**	**Weight (g)**	**Weeks**	**Gender (F/M)**
1	18.2	7	F
2	19.1	7	F
3	21.3	6	F
4	18.4	7	F
5	17.9	7	F
6	19.3	7	F
7	18.4	6	M
8	19.2	6.5	M
9	20.2	7.5	M
10	19.6	7	M
11	18.6	7	M
12	17.8	6.5	M
13	19.6	7	M
14	20.1	6	F
15	18.9	6	M
16	19.8	6	F
17	17.9	7	M
18	19.5	7	M
19	19.3	6.5	F
20	18.2	6	F
*P*-value	>0.05	>0.05	>0.05

### Ultrasound screening results

**Figure 1:**
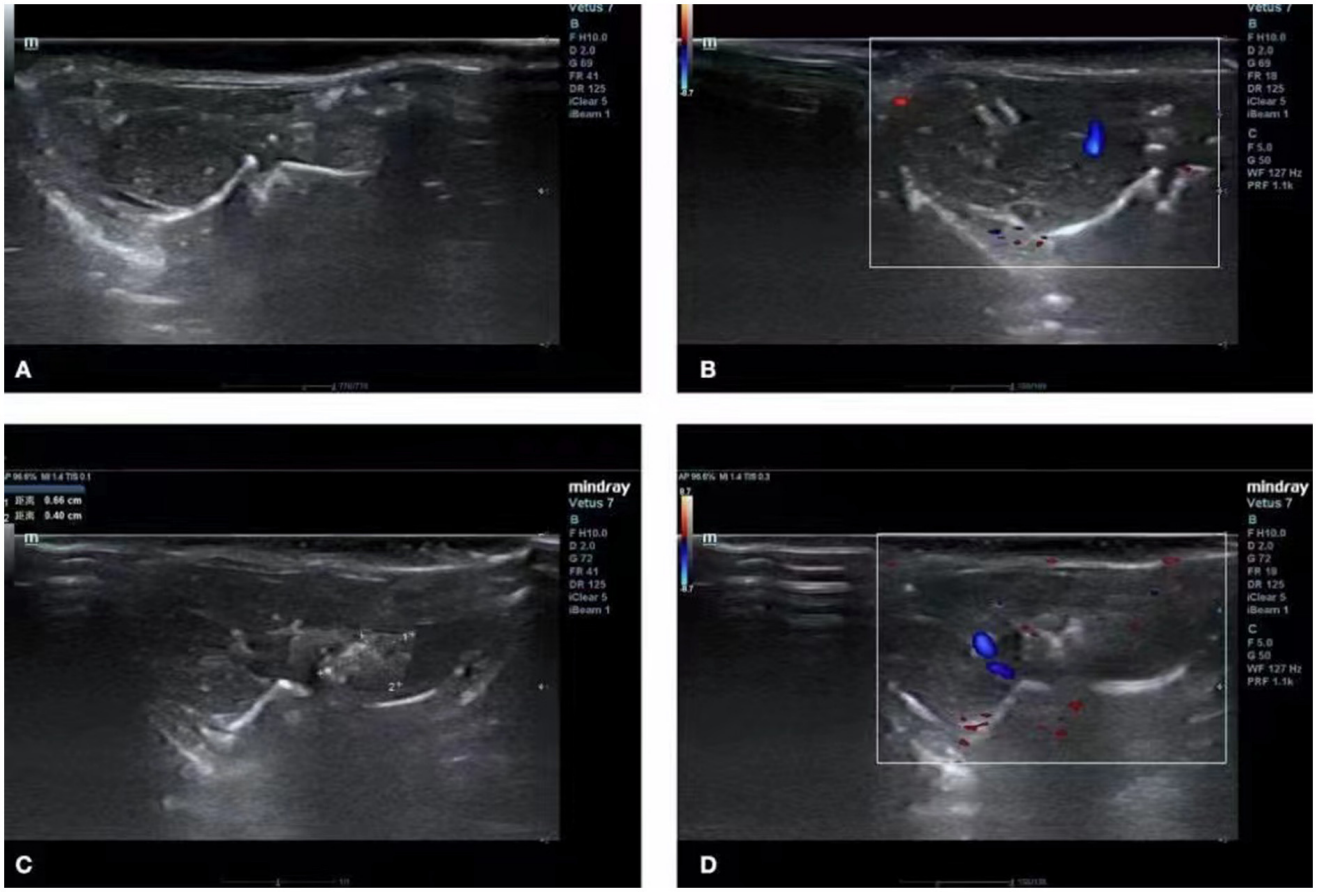
Ultrasound image of the liver. **(A,B)** Ultrasound images of normal livers of mice in the control group. **(C,D)** Images of a hepatic vesicular acinus of successfully infected mice in the experimental group.

The mice in the control group had normal liver morphology, a uniform echo of liver parenchyma without abnormalities, normal morphology of liver vessels, and normal blood flow (Figures 1A,B). The ultrasound images of the experimental group revealed clear lesions in the left lobe of the liver with clear boundaries, uneven and mixed echoes, and internal blood flow (Figures 1C,D).

### Magnetic resonance imaging results

**Figure 2:**
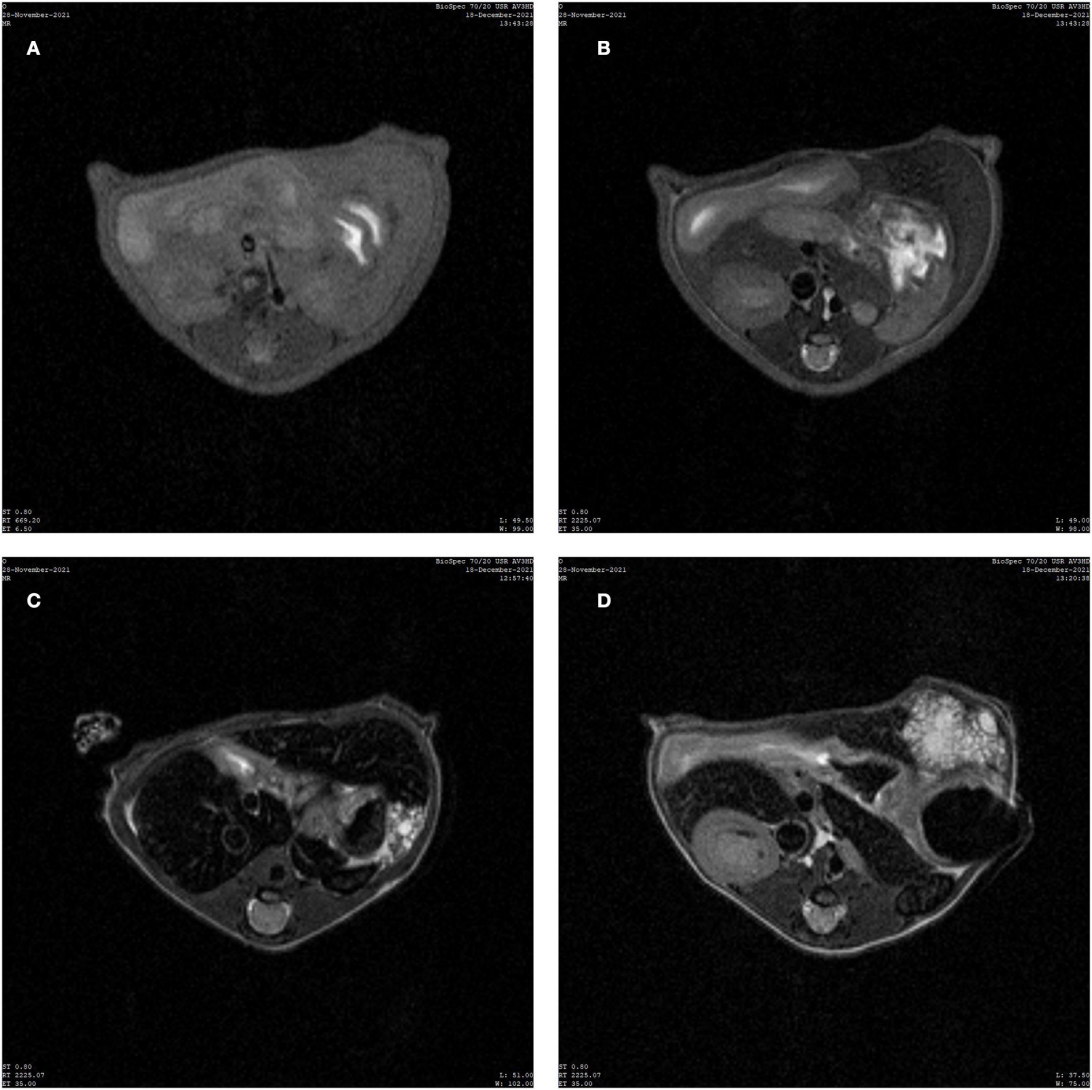
Image of magnetic resonance imaging (MRI). **(A)** MRI T1 weighted image (T1WI) of normal liver of mice in the control group. **(B)** MRI T2 weighted image (T2WI) of normal liver of mice in the control group. **(C,D)** T2WI images of mice successfully infected with hepatic vesicular cercariae.

In the control group, the liver parenchyma signal was uniform, and no abnormal signal was detected inside (Figures 2A,B). The MRIs of the experimental group revealed clear lesions in the left outer lobe of the liver with clear boundaries, mainly with slightly longer transverse relaxation time (T2) signals and multiple vesicles (Figures 2C,D).

### Pathological results of haematoxylin and eosin staining

**Figure 3:**
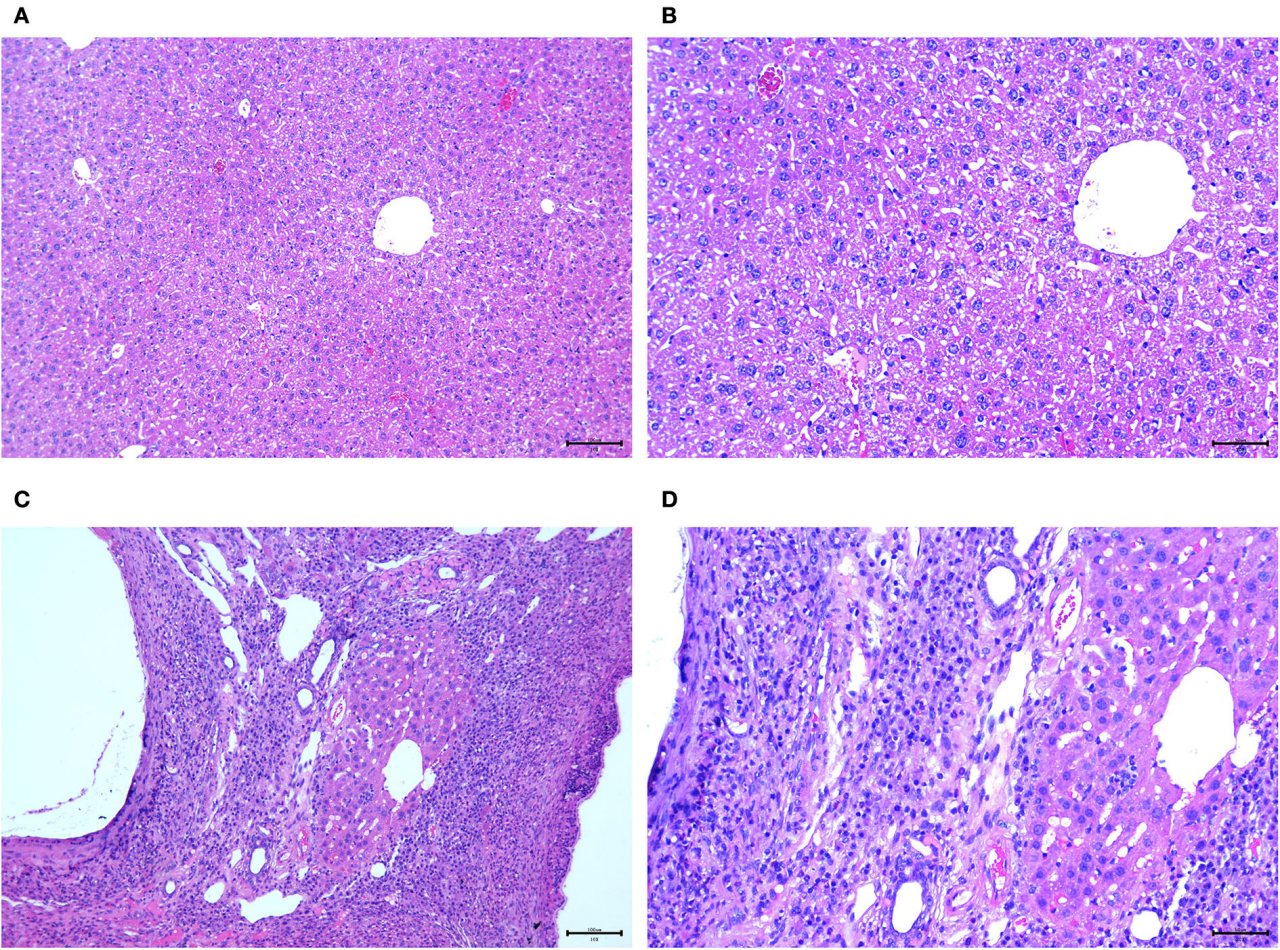
Pathological images. **(A,B)** 100 × and 200 × pathological images of the normal liver of mice in the control group; **(C,D)** 100 × and 200 × pathological images of the diseased liver infected by hepatic vesicular cercariae in the experimental group.

No abnormal changes were observed in the livers of the control group (Figures 3A,B). However, hollow vesicles and fibrous tissue structures were observed in the liver lesions of the experimental group, which increased around the vesicles and inflammatory response areas, including inflammatory cells, microvessels, and fibrotic tissues (Figures 3C,D).

## Discussion

Alveolar echinococcosis is a parasitic disease caused by the larval stage of *Echinococcus*, which is endemic in the northern hemisphere and the western, central, and eastern regions of Asia, with a high prevalence in China (10). Human infection is mainly caused by the ingestion of tapeworm eggs, and the liver is the most common site for primary parasitic lesions. Clinically, parasite invasions are characterized by a long asymptomatic duration (5–15 years on average). At the onset of the symptoms, imaging examinations reveal a large shadow in the liver (11).

The C57 mice were selected for this study since these allowed for feasible ultrasonic and nuclear magnetic detection. The transhepatic portal vein infection of mice is a common and simple surgical method. Using this technique, the mortality rate of the mice was low, and the mice recovered soon after the operation. In the ultrasound test group, the lesions in the liver were clear, with uneven echoes, unclear boundaries, and irregular shapes mixed with strong echoes of different shapes and sizes, followed by wide or palisade tissues. The B-mode ultrasound screening of the HAE model demonstrated high sensitivity and specificity, indicating that it can accurately and reliably screen HAE in experimental animals and can be used to assess the success of modeling (12). The method is safe and fast, avoids scarificing the test model, reduces the pain of the experimental animals, and saves on financial, material, and human resources. The MRI examination results indicated that the T2 signal lesions in the liver were slightly longer and exhibited multiple vesicles (13, 14).

Through a retrospective analysis of the MRI manifestations of different types of HAE, many scholars found that the vesicle-like structure presented as a low-signal intensity round on the MRI T1-weighted image and a high-signal intensity round on the MRI T2-weighted image. The main manifestations of early lesions were multivesicle-like structures without solid internal components. Therefore, the polyvesicle-like structure is considered the main pathological manifestation of early HAE, and early HAE in mice has a similar pathological basis to HAE in humans (15–18). This is consistent with the findings of the current study.

From the H&E results, it was found that all the control group mice exhibited normal liver morphology, no liver lesions, and normal intrahepatic bile ducts and blood flow. Therefore, establishing a mouse HAE model through the intrahepatic portal vein can serve as a good research model for experimental research and provide a better experimental basis for the clinical diagnosis and understanding of HAE.

This study has several limitations. First, the sample size used in this study was small. While we referred to the study by Wang et al. (19) when designing the sample size, the sample size must have been increased to further validate the feasibility of the HAE mouse model. Second, the success rate of infection in our study was 75%. Here, a number of injections may have failed due to the failure of the suspension injection into the intrahepatic portal vein of the mice or due to improper manipulation during the surgery. Third, no comparison with other infection methods was conducted. For example, an open-liver puncture or intrasplenic injection may be a better method of infection. Therefore, future studies need to explore various methods for infecting mice.

## Conclusion

This study used a mouse HAE model to observe and compare liver lesions in the experimental and control group mice in terms of location, size, shape features, and pathological changes, as well as the relationship between the lesions and the adjacent organs. The HAE mouse model was successfully established. This comprehensive study of HAE provides a certain reference value. The HAE C57 mouse model can contribute to the current research in terms of identifying molecules that are related to the formation of the intrahepatic portal vein. In addition, a further procedure could be developed to establish novel models with reference to this study. Finally, the study findings will be useful for the elucidation of the mechanism of intrahepatic portal vein formation and its development.

## Data availability statement

The original contributions presented in the study are included in the article/supplementary material, further inquiries can be directed to the corresponding author.

## Ethics statement

The animal study was reviewed and approved by Ethics Committee of the First Affiliated Hospital of Xinjiang Medical University.

## Author contributions

WL, WT, and WJ: conception, design of the research, and statistical analysis. WT, WJ, JL, ZW, and JW: acquisition of the data, analysis, and interpretation of the data. WT and WJ: writing of the manuscript. WL: critical revision of the manuscript for intellectual content. All authors contributed to the article and approved the submitted version.
